# Incidence of mastoiditis complications in relation to prior community-based antibiotic treatment

**DOI:** 10.1007/s00405-026-10349-y

**Published:** 2026-06-01

**Authors:** Nitzan Sofer, Ayalon Hadar, Gabriel Choucroun, Yehuda Tarnovsky, Ronen Perez, Pierre Attal, Chanan Shaul

**Affiliations:** Department of Otolaryngology, Head and Neck Surgery, The Faculty of Medicine, Shaare Zedek Medical Center, Hebrew University, POB 3235, Jerusalem, 91031 Israel

**Keywords:** Acute otitis media, Acute mastoiditis, Subperiosteal abscess, Epidural abscess, Sigmoid sinus thrombosis

## Abstract

**Objectives:**

To evaluate the association between prior community-based antibiotic treatment for acute otitis media (AOM) and the incidence and severity of complications in children hospitalized with acute mastoiditis (AM), noting that this design does not address whether prior antibiotic treatment prevents AM development.

**Methods:**

This retrospective cohort study analyzed medical records of 522 children (age < 15 years) diagnosed with AM at a tertiary medical center between 2015 and 2025. Children were categorized by prior antibiotic exposure: those receiving antibiotics for > 48 h before hospitalization versus those receiving < 48 h or none. The primary outcome was the presence of complicated AM, defined as extracranial (subperiosteal abscess) or intracranial (epidural abscess, sigmoid sinus thrombosis, brain abscess, or meningitis) complications.

**Results:**

Of 522 children, 110 (21%) received prior antibiotics and 412 (79%) did not. While the composite complication rate was higher in the prior treatment group (54.5% vs. 38.6%, unadjusted OR = 1.91, *p* = 0.003), prior antibiotic treatment was not significantly associated with specific complication types, including subperiosteal abscess (adjusted OR = 0.67, *p* = 0.37) or intracranial complications (adjusted OR = 1.74, *p* = 0.13). Inflammatory markers, hospitalization length, imaging use, and surgical intervention rates were comparable between groups. Fusobacterium necrophorum in blood cultures was significantly associated with complications regardless of prior antibiotic exposure.

**Conclusions:**

Among children hospitalized with AM, prior antibiotic treatment was not associated with increased rates of specific complications. The difference in composite outcomes likely reflects selection bias rather than a true treatment effect. Causal inference is limited by confounding by indication and unmeasured baseline severity differences.

## Introduction

Acute otitis media (AOM) is among the most prevalent pediatric infectious diseases worldwide, affecting up to 84% of children by the age of three and representing a leading cause of antibiotic prescriptions in childhood [1, 2]. The condition accounts for millions of healthcare visits annually and imposes a significant public health burden due to its high incidence, healthcare utilization, and risk of complications [3–5].

One of the most serious complications of AOM is acute mastoiditis (AM), resulting from the spread of infection from the middle ear to the mastoid air cells. Although the introduction of antibiotics in the mid-20th century dramatically reduced the incidence and mortality associated with AM, it remains a potentially life-threatening condition requiring prompt diagnosis and management [6, 7]. The clinical presentation of AM frequently includes postauricular swelling, erythema, auricular protrusion, fever, and systemic symptoms [8]. If not promptly treated, the infection can progress to severe extracranial and intracranial complications, including subperiosteal abscess, facial nerve palsy, meningitis, intracranial abscess, or sigmoid sinus thrombosis [6, 8, 9]. Current estimates suggest an incidence of 1.2–4.2 cases per 100,000 children per year in developed countries, although significant geographical and temporal variations exist [3, 4, 6].

In recent decades, the management of uncomplicated AOM has shifted toward a more conservative approach, favoring delayed antibiotic prescribing or watchful waiting to mitigate overuse and curb antimicrobial resistance [7]. This paradigm shift represents a fundamental change in clinical practice, transitioning from immediate antibiotic treatment to a more targeted approach based on symptom severity, patient age, and risk factors. The strategy is supported by evidence that most AOM episodes resolve spontaneously without antibiotics, with approximately 80% resolving within 2–3 days and 90% within 1 week without intervention [9–11]. The American Academy of Pediatrics (AAP) guidelines recommend delayed antibiotic prescribing in specific cases: (a) AOM without signs of severity in unilateral cases without otorrhea in children 6 months to 2 years, or (b) unilateral or bilateral cases without otorrhea in children older than 2 years [12].

However, there is ongoing debate regarding whether this conservative approach may be associated with increased AM development. The clinical dilemma is complex, balancing the benefits of reduced antibiotic exposure against the potential risk of serious complications. An extensive retrospective cohort study in the United Kingdom demonstrated an association between antibiotic use and reduced AM risk, with antibiotics approximately halving the risk of AM development, although the number needed to treat to prevent a single case was over 4,000, highlighting the limitations of prophylactic antibiotic use from both clinical and economic perspectives [7]. Other studies, however, have found no clear association between delayed antibiotic treatment and higher rates of mastoiditis, creating uncertainty in clinical decision-making [13–15].

Moreover, the potential association between delayed treatment and the severity and complication profile of AM once it occurs has been less thoroughly investigated. While previous research has evaluated how prior antibiotic exposure may alter microbiological patterns and resistance profiles in mastoiditis [15, 16], the relationship between community-based antibiotic treatment and the severity of AM at presentation remains incompletely understood.

The present study addresses precisely this latter question: not whether antibiotics prevent AM development, but whether prior antibiotic exposure influences the complication profile in children who have already developed AM. It is important to note that the present study cannot determine whether delayed antibiotic treatment for AOM increases the risk of AM development, as our entry point is children who already have AM. Instead, the study aims to address a critical knowledge gap by evaluating whether prior antibiotic treatment is associated with different presentations and complication profiles in children hospitalized with AM. Specifically, we examine the relationship between antecedent antibiotic exposure and the incidence of extracranial and intracranial complications, as well as differences in fever, inflammatory markers, and microbiological findings. By clarifying these associations, the study aims to inform evidence-based recommendations for prescribing antibiotics for AOM and for the management of this potentially severe complication.

## Methods

### Study design and setting

A retrospective cohort study was conducted analyzing medical records of children diagnosed with acute mastoiditis (AM) who presented to our tertiary medical center pediatric emergency department between 2015 and 2025. The study was approved by the institutional review board (approval #0205-23-SZMC), and informed consent was waived due to its retrospective nature and the use of de-identified data.

### Study population

**Fig. 1:**
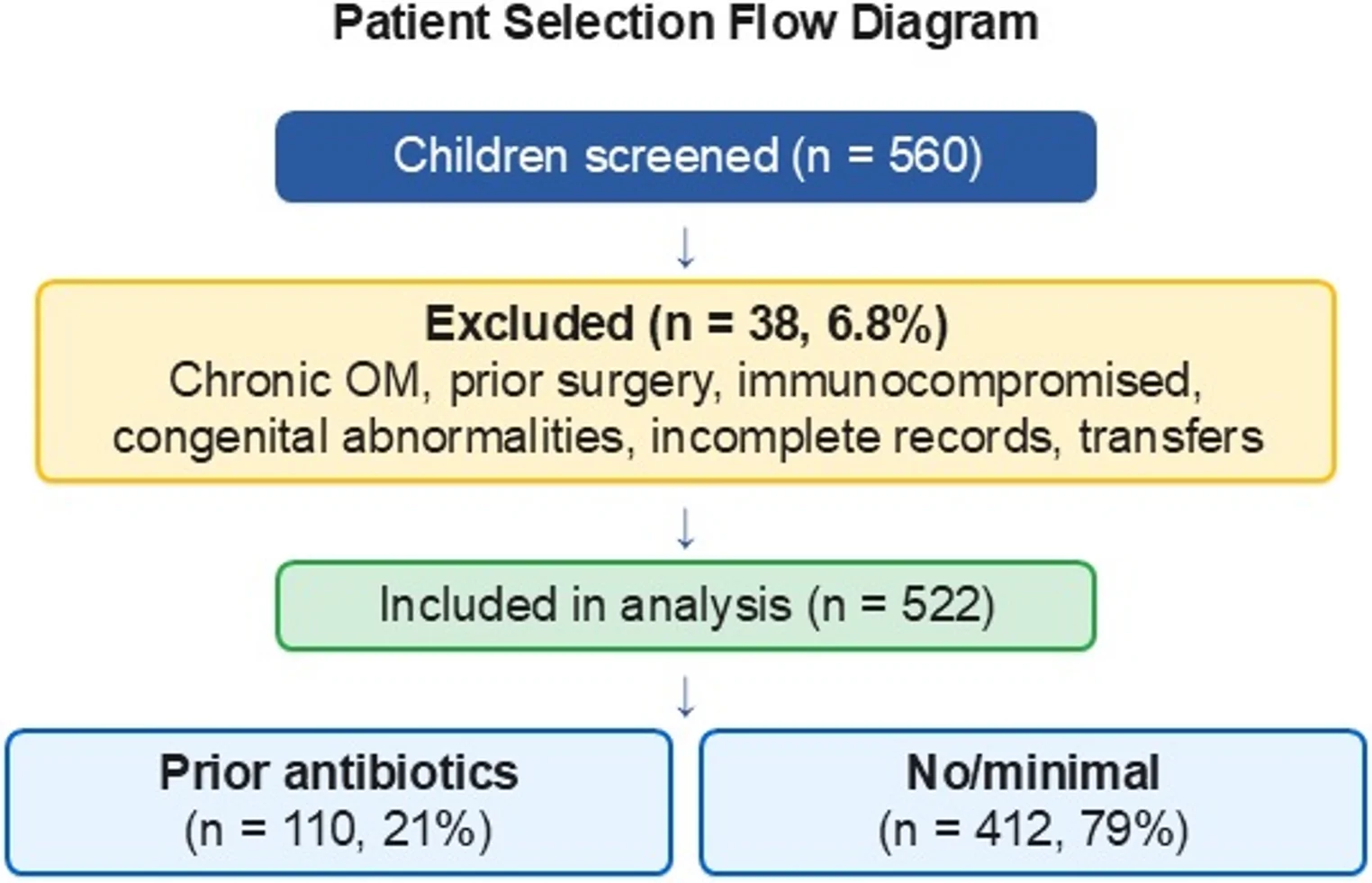
Flow diagram of patient inclusion and exclusion. Flow chart showing the process of patient selection, including total number of potential cases identified, exclusion criteria applied with reasons, and final number of patients included in each study group

The study included all children under 15 years of age who were diagnosed with AM during the study period. AM was defined based on clinical presentation, including postauricular swelling, erythema, auricular protrusion, fever, and radiological evidence when available. Cases were identified through electronic health record searches using relevant ICD-10 codes and clinical keywords. The research team reviewed all potential cases to confirm the diagnosis and ensure inclusion criteria were met. Figure 1 presents a flow diagram of patient inclusion and exclusion.

Exclusion criteria included children with chronic otitis media, previous mastoid surgery, immunocompromised status, congenital ear abnormalities, or incomplete medical records that prevented adequate data collection. Children transferred from other hospitals who were receiving ongoing antibiotic treatment were also excluded to ensure accurate assessment of community-based antibiotic exposure.

### Data collection

All medical records were thoroughly reviewed from admission through discharge by trained research personnel using a standardized data collection form. The following data were systematically collected for each patient: demographic details including age, sex, and month of admission; clinical presentation including vital signs, physical examination findings, and symptom duration; laboratory investigations including complete blood count with differential, C-reactive protein (CRP) levels, and blood cultures when performed; imaging studies including computed tomography (CT) or magnetic resonance imaging (MRI) when available; microbiological data from ear, abscess, and blood cultures; treatment modalities including intravenous antibiotics, surgical drainage procedures, and mastoidectomy; and clinical outcomes including length of hospitalization, complications, and follow-up data.

### Exposure and outcome definitions

For the purpose of this study, prior community-based antibiotic treatment was defined as antibiotic therapy for more than 2 days before hospitalization for AM. Patients were categorized into two groups: those who received antibiotic treatment for more than 48 h (defined as having received prior antibiotic treatment) and those who did not receive antibiotic treatment at all, or received it for 48 h or less (defined as not having received prior antibiotic treatment).

This dichotomous classification was chosen based on clinical guidelines that typically recommend initial observation for 48–72 h in uncomplicated cases of acute otitis media, making the 2-day threshold clinically meaningful for distinguishing between conservative management and established antibiotic therapy [12]. Furthermore, this 48-hour threshold helps ensure that antibiotic treatment preceded AM development, since community physicians prescribing oral therapy had evaluated the child without finding signs of mastoiditis; children presenting with mastoiditis signs would have been referred directly to the emergency department. Antibiotic courses of less than 48 h are also unlikely to exert meaningful bacteriological effects; this threshold therefore distinguishes patients who received clinically consequential antibiotic exposure from those who did not.

Data on prior antibiotic treatment were obtained from three complementary sources: the referral letter from the community physician, parental report at admission, and review of the electronic community medical records, where available.

Sensitivity analyses were further conducted using alternative exposure definitions. First, we examined a more stringent 72-hour (3-day) cutoff, classifying patients as exposed only if they received antibiotics for more than 3 days prior to hospitalization. Second, among patients with available duration data (*n* = 99), we analyzed antibiotic duration as a continuous variable to assess potential dose-response relationships. Duration was examined both as days of treatment and categorized into groups (3–4 days, 5–7 days, > 7 days).

The primary outcome was defined as complicated AM, characterized by extracranial complications (subperiosteal abscess requiring surgical drainage) or intracranial complications (epidural abscess, brain abscess, sigmoid sinus thrombosis, or meningitis). Facial nerve palsy was not observed in our cohort and was therefore not included as an outcome.

While “any complication” served as the primary composite outcome, we recognized that extracranial and intracranial complications represent clinically distinct entities with different severities and management implications. Therefore, complications were analyzed both as a composite endpoint and stratified by type (extracranial vs. intracranial) to avoid over-reliance on the composite outcome alone. Secondary outcomes included fever magnitude at presentation, inflammatory marker levels (white blood cell count (WBC), neutrophil count, and C-reactive protein (CRP)), length of hospital stay, use of imaging studies (CT or MRI), need for surgical intervention, and microbiological findings from various culture sites.

Imaging studies (CT or MRI) at our institution were not performed routinely for all AM cases; they were obtained selectively based on clinical criteria. Specifically, imaging was performed when patients showed no clinical improvement (e.g., persistent high fever) after 48 h of intravenous antibiotic therapy. When intracranial complications were demonstrated on imaging, surgical intervention was performed. It should be noted that imaging use and surgical intervention rates, although analyzed as outcomes, are influenced by institutional protocols and clinician practice patterns.

### Statistical analysis

Statistical analysis was conducted using SPSS software (IBM^®^ SPSS^®^ Statistics, version 25, Chicago, IL, USA). Descriptive statistics were used to characterize the study population, with continuous variables presented as means with standard deviations and categorical variables as frequencies and percentages. The distribution of continuous variables was assessed using the Kolmogorov-Smirnov test to determine appropriate statistical tests.

Comparisons between the two groups (those with prior antibiotic treatment versus those without) were performed using various statistical methods. For categorical variables, the chi-square test for independence with continuity correction was employed. For continuous variables, Student’s t-test or Mann-Whitney U test were used as appropriate. Odds ratios with 95% confidence intervals were calculated for binary outcomes to describe associations, not causal relationships.

Additionally, the duration of antibiotic treatment was analyzed as a continuous variable to assess potential dose-response relationships between length of prior treatment and study outcomes. Correlation analyses were performed to examine associations between treatment duration and continuous outcome measures such as fever magnitude and inflammatory marker levels.

All statistical tests were two-tailed, and a p-value less than 0.05 was considered statistically significant. The observational nature of this study limits causal inference. We present associations rather than causal relationships throughout the results and discussion.

To address potential confounding, multivariable logistic regression models were constructed. Model 1 (*n* = 487) predicted any complications, adjusting for age, sex, fever at presentation, and white blood cell count. Covariates were selected a priori: age, fever, and white blood cell count as clinical markers of disease severity at presentation, and sex as a standard demographic covariate included to account for potential group imbalances. Models 2 and 3 examined specific complication types among patients with complications (*n* = 202): Model 2 predicted extracranial complications (subperiosteal abscess) and Model 3 predicted intracranial complications (meningitis, epidural abscess, brain abscess, sigmoid sinus thrombosis). Note that 15% of patients with complications had both extracranial and intracranial involvement. Results are presented as adjusted odds ratios (aOR) with 95% confidence intervals.

Missing data were handled using complete case analysis (listwise deletion), whereby patients with missing values for any covariate in a given model were excluded from that analysis. To assess potential selection bias from missing data, we compared baseline characteristics and complication rates between patients with complete versus missing data for each model.

## Results

Between 2015 and 2025, 522 children were diagnosed with AM. There were 277 boys (53%) and 245 girls (47%). The average age was 2.4 (SD 2.3) years (age range between 2 months and 14 years). There were 412 children (79%) in the non-treatment group and 110 children (21%) in the prior antibiotic treatment group, the vast majority of whom received amoxicillin or amoxicillin-clavulanic acid. This division was the basis for all comparisons. Demographic details and the initial presentation, including inflammatory markers, are shown in Table 1.

**Table 1:** Demographic details and presentation of acute mastoiditis cases. Demographic characteristics of acute mastoiditis cases, fever, and mean laboratory values. WBC- White Blood Count. CRP- C-reactive protein. Percentages for categorical variables are calculated relative to the total number of patients in each group (prior treatment, *n* = 110; non-treatment, *n* = 412). Comparisons of continuous variables (age, fever, and inflammatory markers) were conducted using Student’s t-test. Categorical variables (sex) were analyzed using the Pearson chi-square test

	Prior treatment	Non-treatment	*p*-value
Number	110	412	
Age, years in median [IQR, Q1,Q3]	1.65 [2.58,1.1,3.68]	1.3 [2.0,0.8,2.8]	0.088
Gender M/F	60/50	217/195	0.726
Fever (°C )	37.5	37.7	0.036
WBC (Nx10^9^)	17.1	17.9	0.262
Neutrophils (%)	52.93	55.5	0.144
CRP (mg/dl)	9.8	10.7	0.424

The primary outcome, complicated AM, occurred in 219 children (41.9% of the cohort). Extracranial complications occurred in 186 children (35.6%), with subperiosteal abscess being the sole extracranial complication type. Intracranial complications occurred in 62 children (11.9%), including epidural abscess in 34 (6.5%), sigmoid sinus thrombosis in 39 (7.5%), and meningitis in 3 (0.6%); note that some children had more than one complication. Complication rates were higher in the prior treatment group (60/110, 54.5%) compared to the non-treatment group (159/412, 38.6%), with an unadjusted OR of 1.91 (*p* = 0.003). However, this association was driven by the composite outcome, which combines clinically distinct entities. When examined separately, prior antibiotic treatment was not significantly associated with extracranial complications (subperiosteal abscess: adjusted OR = 0.67, *p* = 0.37) or intracranial complications (adjusted OR = 1.74, *p* = 0.13). Demographic characteristics and inflammatory markers by group are presented in Table 1. Complication rates, imaging use, and surgical intervention are presented in Table 2.

**Table 2:** Correlation between prior antibiotic treatment and various outcomes. Complications, need for imaging, surgical management, and length of hospitalization were assessed in patients who received or did not receive prior antibiotic treatment. In our institution, imaging (CT/MRI) was performed selectively in patients who did not show clinical improvement after 48 h of IV antibiotics, and surgical intervention was performed for demonstrated intracranial complications. These rates reflect institutional protocols and practice patterns, as well as disease severity. Some patients presented with more than one complication; therefore, the total number of complications exceeds the number of patients (*n* = 219). Comparisons of complications, imaging, and surgery were conducted using the Pearson Chi-square test. Length of Hospitalization was analyzed using the Mann–Whitney U test

	Prior treatment	Non-treatment	total	*p*-value
Number	110	412	522	
Complications:				
Subperiosteal abscess	49 (81.7%)	137 (86.2%)	186	0.407
Epidural abscess	11 (18.3%)	23 (14.5%)	34	0.481
Sigmoid vein thrombosis	13 (21.7%)	26 (16.4%)	39	0.359
Meningitis	0 (0.0%)	3 (1.9%)	3	0.564
Need for Imaging	41(37.3%)	116 (28.2%)	157	0.064
Surgery	28 (25.4%)	77(18.7%)	105	0.114
Length of Hospitalization. Mean ± SD	7.86 ± 5.95	7.0 ± 5.08	-	0.128

No significant association was found between prior antibiotic use and inflammatory markers on admission; WBC, neutrophils, and CRP levels were similar in both groups. No significant differences were found between the groups in hospitalization length. Rates of imaging use and surgical intervention were also comparable between groups, though these outcomes reflect institutional protocols (imaging performed for lack of clinical improvement after 48 h of IV antibiotics; surgical intervention for demonstrated intracranial complications) as well as disease severity (Table 2). The type of antibiotics administered before hospitalization was not associated with complication rates (*p* = 0.107).

The distribution of pathogens across both groups is shown in Table 3. While in the ear and abscess cultures, Streptococcus pyogenes was the predominant pathogen; in the positive blood cultures, Fusobacterium necrophorum was the predominant pathogen. Some differences in pathogen prevalence were observed between the groups. Haemophilus influenzae was identified among the top three pathogens only in the non-treatment group (11.5% of ear cultures), whereas Staphylococcus spp. appeared more consistently in the prior-treatment group, particularly in ear and abscess cultures. In blood cultures, Fusobacterium necrophorum was observed in 21% of the non-treatment group versus 15% of the prior treatment group (*p* = 0.532). A higher proportion of negative cultures was noted after antibiotic exposure, suggesting a reduced culture yield; however, the dominant pathogens—Streptococcus beta-hemolytic Group A and Streptococcus pneumoniae—remained similar across groups.

**Table 3:** Ear, abscess, and blood culture results of acute mastoiditis. Rates of positive cultures in the prior treatment group (110 children) and non-treatment group (412), stratified by Source (Ear, Abscess, and Blood), followed by the prevalence of the most common pathogens in any group. Culture positivity rates are expressed as percentages of all patients in each group. Pathogen prevalence is expressed as a percentage of positive cultures from each source. No formal statistical comparison was performed for individual pathogen prevalence

	Prior treatment	Non-treatment	*p*-value
Positive culture	91 (82.7%(	395 (95.9%(	0.000
Ear (*n* = 467)	68 (61.8%)	309 (75.0%)	0.006
Most common	Streptococcus beta-hemolytic Group A (19, 21%)	Streptococcus beta-hemolytic Group A (125, 33.6%)	
2nd common	Streptococcus pneumoniae (14, 16%)	Streptococcus pneumoniae (63, 17%)	
3rd common	Staphylococcus spp. (10, 11%)	Haemophilus influenza (43, 11.5%)	
Abscess (*n* = 120)	14 )12.7%)	64 )15.5%)	0.467
Most common	Fusobacterium necrophorum (7, 23%)	Streptococcus beta-hemolytic Group A (22, 25%)	
2nd common	Streptococcus pneumoniae (4,13%)	Streptococcus pneumoniae (22, 25%)	
3rd common	Staphylococcus spp. (2, 6%)	Fusobacterium necrophorum (9, 10%)	
Blood (*n* = 100)	9 )8.2%(	24 (5.8%)	0.368
Most common	Streptococcus spp. (4, 20%)	Fusobacterium necrophorum (17, 21%)	
2nd common	Fusobacterium necrophorum (3, 15%)	Streptococcus spp. (3, 4%)	
3rd common	Others (2, 10%)	Staphylococcus spp. (2, 2.5%)	

To evaluate the associations between prior antibiotic treatment and bacterial distributions, as well as between specific pathogens and complication rates, analyses were performed for each pathogen across culture types. These comparisons are presented in Table 4.

**Table 4:** Correlation between prior antibiotic treatment, various pathogens, and complications. Analysis of the correlation between specific pathogens in different cultures, prior antibiotic treatment, and the subsequent risk of clinical complications, presented as unadjusted odds ratios with corresponding 95% Confidence Intervals. Odds ratios are unadjusted and were calculated using the Pearson chi-square test. The left column presents the association between prior antibiotic treatment and each pathogen. The right column presents the association between each pathogen and the presence of complications

Prior antibiotic treatment	OR (CI)	*p*-value	pathogen	OR (CI)	p-value	Presence of complication
	0.5 (0.31–0.9)	0.018	Streptococcus Group A (ear)	0.69 (0.46–1.04)	0.078	
	0.16 (0.04–0.71)	0.007	Streptococcus Group A (**abscess**)	1.44 (0.38–5.47)	0.587	
	0.85 (0.45–1.58)	0.599	Streptococcus pneumoniae (**ear**)	0.86 (0.52–1.43)	0.574	
	0.45 (0.14–1.43)	0.169	Streptococcus pneumoniae (**abscess**)	0.55 (0.17–1.77)	0.318	
	0.7 (0.32–1.55)	0.377	Haemophilus influenza (**ear**)	0.88 (0.48–1.60)	0.687	
	1.1 (0.52–2.29)	0.810	Staphylococcus spp. (**ear**)	0.72 (0.38–1.36)	0.306	
	2.59 (0.87–7.7)	0.079	Fusobacterium necrophorum (**abscess**)	1.01 (0.22–5.31)	0.916	
	0.65 (0.17–2.5)	0.532	Fusobacterium necrophorum (**blood**)	24.43 (3.11-191.44)	0.00	
	1.91 (1.13–3.21)	**0.014**	No growth **(ear)**	1.73 (1.09–2.74)	0.019	
	3.11 (1.34–7.24)	0.007	No growth (**abscess**)	1.21 (0.39–3.76)	0.736	

In this analysis, prior treatment was associated with increased culture negativity, as expected given antibiotics’ effects on bacterial growth. Among specific pathogens, the most notable findings were the association of Fusobacterium necrophorum in blood cultures with complications. Given the large number of pathogen-specific comparisons performed (across multiple pathogens and culture types), individual pathogen associations should be interpreted cautiously due to potential type I error from multiple comparisons.

### Multivariable analysis of complications

To address potential confounding, we conducted multivariable logistic regression analyses adjusting for age, sex, fever, and white blood cell count (Table 5).

**Table 5:** Multivariable logistic regression models predicting acute mastoiditis complications. Model 1 includes all patients with complete covariate data. Models 2 and 3 examine specific complication types among patients who had any complication (*n* = 202 with complete data). Extracranial complications refer to subperiosteal abscess. Intracranial complications include meningitis, epidural abscess, brain abscess, and sigmoid sinus thrombosis. All models adjust for age, sex, fever at presentation, and white blood cell count. Model 1 includes all patients with complete covariate data (*n* = 487). Models 2 and 3 examine specific complication types among patients with any complication (*n* = 202). Note that 15% of patients with complications had both extracranial and intracranial complications. aOR = adjusted odds ratio; CI = confidence interval; WBC = white blood cell count. **Bold indicates** ***p*** < 0.05

Variable	Model 1: Any complications (*n* = 487)	Model 2: Extracranial complications† (*n* = 202)	Model 3: Intracranial complications† (*n* = 202)
	aOR (95% CI), *p*-value	aOR (95% CI), *p*-value	aOR (95% CI), *p*-value
Prior Antibiotics	**2.17 (1.38–3.41)**, ***p*** = 0.001	0.67 (0.28–1.62), *p* = 0.37	1.74 (0.85–3.55), *p* = 0.13
Age (per year)	1.03 (0.94–1.12), *p* = 0.58	**0.81 (0.70–0.94)**, ***p*** = 0.005	**1.16 (1.01–1.33)**, ***p*** = 0.03
Sex (female)	0.99 (0.68–1.44), *p* = 0.95	0.73 (0.32–1.67), *p* = 0.46	1.07 (0.56–2.04), *p* = 0.85
Fever (per °C)	1.16 (0.95–1.42), *p* = 0.16	1.05 (0.67–1.65), *p* = 0.84	**1.62 (1.15–2.28)**, ***p*** = 0.006
WBC (per K/µL)	**1.04 (1.01–1.08)**, ***p*** = 0.005	0.97 (0.92–1.03), *p* = 0.30	1.02 (0.98–1.07), *p* = 0.36

In the primary model predicting any complications (a composite of extracranial and intracranial) (*n* = 487 with complete data), prior antibiotic treatment remained strongly associated with the composite outcome after adjustment (aOR = 2.17, 95% CI: 1.38–3.41, *p* = 0.001). However, this composite association masked important differences by complication type. Notably, the adjusted odds ratio was higher than the unadjusted odds ratio (1.91), indicating the association strengthened after adjustment. White blood cell count was also a significant predictor (aOR = 1.04 per K/µL, *p* = 0.005).

Among patients with complications (*n* = 202), the pattern differed by type of complication. Prior antibiotic treatment was not significantly associated with extracranial complications (subperiosteal abscess: aOR = 0.67, 95% CI: 0.28–1.62, *p* = 0.37). For intracranial complications, there was a non-significant trend toward higher risk with prior antibiotics (aOR = 1.74, 95% CI: 0.85–3.55, *p* = 0.13). Other predictors included younger age for extracranial complications (aOR = 0.81 per year, *p* = 0.005), older age for intracranial complications (aOR = 1.16 per year, *p* = 0.03), and higher fever for intracranial complications (aOR = 1.62 per °C, *p* = 0.006).

Importantly, 15% of patients with complications had both extracranial and intracranial involvement. Baseline inflammatory markers were similar in the prior antibiotic group compared to the non-treatment group. While fever was statistically lower in the prior antibiotic group (37.54 °C vs. 37.75 °C, *p* = 0.036), this 0.2 °C difference is unlikely to be clinically meaningful.

### Sensitivity analyses of antibiotic exposure definition

In the sensitivity analysis using the 72-hour cutoff, 78 patients (15%) were classified as having received prior antibiotics. In multivariable analysis, the association with complications was even stronger (adjusted OR = 3.02, 95% CI: 1.78–5.13, *p* < 0.001) than with the 48-hour cutoff (adjusted OR = 2.17, *p* = 0.001).

Among patients with available antibiotic duration data (*n* = 99, representing 90% of those who received antibiotics), the mean duration was 6.1 days (SD = 2.7, range 3–14 days). When analyzing duration as a continuous variable in multivariable models (*n* = 91 with complete covariate data), each additional day of antibiotic treatment was not significantly associated with complications (adjusted OR = 1.02 per day, 95% CI: 0.87–1.20, *p* = 0.79). Analysis by duration categories (3–4 days, 5–7 days, > 7 days) showed complication rates of 54.5%, 52.5%, and 66.7%, respectively, with no clear dose-response pattern. These analyses were limited by the small sample size of patients with duration data and represent only those who received prolonged antibiotic courses.

Missing data rates varied by variable and exposure group. Laboratory data were missing for: fever in 28 patients (5.4%), white blood cell count in 7 patients (1.3%), neutrophils in 8 patients (1.5%), and C-reactive protein in 79 patients (15.1%). Importantly, C-reactive protein data were more frequently missing in the prior antibiotic group (21.8%) compared to the no antibiotic group (13.3%, *p* = 0.04), suggesting differential missingness between exposure groups. White blood cell count was also more frequently missing in the prior antibiotic group, though this did not reach statistical significance (3.6% vs. 0.7%, *p* = 0.06). For the primary multivariable Model 1 analysis, 35 patients (6.7%) were excluded due to missing covariate data (primarily fever and white blood cell count), resulting in an analytic sample of 487 patients. Patients excluded due to missing data had numerically higher complication rates (17 of 35, 48.6%) compared to those included in the model (202 of 487, 41.5%), though this difference was not statistically significant (*p* = 0.43, chi-square test).

## Discussion

This study evaluated the association between prior community-based antibiotic therapy and the severity of complications among children hospitalized with AM. An important finding of our study is that children who did not receive early antibiotic treatment or received only short-course treatment (up to 48 h) did not have higher rates of specific complications during subsequent hospitalizations for AM. However, this study cannot address whether delayed antibiotic treatment affects the risk of developing AMs from AOM in the first place, as our entry point is children who already had AM [11, 15, 17–19].

Before interpreting these findings, it is important to acknowledge two inherent limitations of the retrospective design: the potential for indication bias, whereby children selected for antibiotic treatment may have had more severe initial disease, and the general constraints of retrospective data collection, including incomplete documentation and reliance on parental recall.

For many years, there has been debate regarding the risks and benefits of conservative approaches to AOM management. A primary concern has been that delaying or withholding antibiotic therapy might be associated with increased risk of AM development and more severe complications. Our findings are consistent with the existing evidence on conservative management. We found no association between the lack of early antibiotic treatment and higher rates of specific complications, such as subperiosteal abscesses or intracranial complications, upon hospitalization. This finding is consistent with previous studies that have found no clear association between delayed antibiotic treatment and complication severity in mastoiditis [13, 14, 17].

However, we observed that children in the prior treatment group exhibited a higher rate of the composite outcome “any complication” (unadjusted OR 1.9, *p* = 0.003). Importantly, this composite outcome combines clinically heterogeneous entities—extracranial complications (primarily subperiosteal abscess, which is common and generally manageable) and intracranial complications (which are rarer but more severe). To address potential confounding, we conducted multivariable logistic regression analyses adjusting for age, sex, fever, and white blood cell count (Table 5). After adjustment, the association strengthened (adjusted OR = 2.17, 95% CI: 1.38–3.41, *p* = 0.001), and children who received prior antibiotics presented with slightly lower fever at hospital admission (37.54 °C vs. 37.75 °C, *p* = 0.036—though this 0.2 °C difference, while statistically significant, is unlikely to be clinically meaningful)—patterns opposite to what simple confounding by indication would predict. Importantly, when examining specific complication types among patients with complications, prior antibiotics showed no significant association with either extracranial (adjusted OR = 0.67, *p* = 0.37) or intracranial (adjusted OR = 1.74, *p* = 0.13) complications, although 15% of patients had both. This pattern underscores that statistical adjustment cannot overcome selection on unmeasured variables that drive both treatment decisions and outcomes.

When we stratified by complication type rather than relying on the composite outcome, a more nuanced picture emerged. Prior antibiotic treatment showed no significant association with either extracranial complications (adjusted OR = 0.67, *p* = 0.37) or intracranial complications (adjusted OR = 1.74, *p* = 0.13) when analyzed separately. This discordance between the composite outcome (significant association, OR = 2.17) and the stratified analyses (no significant associations) likely reflects the complexity of selection bias and highlights the limitations of composite outcomes that combine clinically distinct entities. The composite association may be driven by unmeasured factors related to disease severity rather than by specific effects on particular types of complications.

Our primary analysis used a 48-hour cutoff, consistent with clinical guidelines recommending observation for 48–72 h in uncomplicated AOM. To assess the robustness of our findings, we conducted sensitivity analyses using a more stringent 72-hour (3-day) cutoff. Even among children who received oral antibiotics for more than 3 days, complication rates remained high (adjusted OR = 3.02), indicating that prolonged oral antibiotic treatment did not prevent complications once AM developed. This finding suggests that community-based oral antibiotics, even when administered for several days, may not prevent complications in children whose disease progresses to AM.

The strengthening of the association with a more stringent cutoff (adjusted OR = 3.02 at 72 h vs. 2.17 at 48 h) further supports that children requiring longer antibiotic courses likely represent a subset with inherently more severe or treatment-resistant disease from the outset. Among patients with detailed duration data (*n* = 91), we found no dose-response relationship between antibiotic duration and complication risk (adjusted OR = 1.02 per day, *p* = 0.79), suggesting that once a child’s disease trajectory is toward AM, extending oral antibiotic duration does not substantially reduce complication severity.

Importantly, our core finding remains: among children who developed AM, complication types, severity markers, and clinical course were similar regardless of whether prior antibiotic exposure was defined at 48–72 h. These findings are robust across different exposure definitions.

It is important to acknowledge that observational studies such as ours cannot establish causality due to the absence of randomization and the presence of unmeasured confounding. While we found no associations between prior antibiotic use and certain outcomes, these findings should not be interpreted as causal. More advanced analytical approaches, such as propensity score methods, inverse probability of treatment weighting, or doubly robust models, could help mitigate confounding in future studies, though they cannot fully eliminate bias inherent in observational designs.

Regarding antibiotic type, no association was observed between amoxicillin-clavulanate and penicillin alone and complication incidence. This finding aligns with existing AOM treatment guidelines [12].

In the microbiological component of our study, we examined the association between prior antibiotic treatment and the prevalence of different pathogens. Analysis showed that prior antibiotic treatment was not associated with substantial shifts in the prevalence of dominant bacteria in either ear or abscess cultures. However, some differences in the relative abundance of specific pathogens were noted. These findings suggest that prior antibiotic exposure may not markedly alter the dominant bacterial profile in AM, which contrasts with the hypothesis of pathogen replacement under selective pressures [20, 21]. These microbiological findings complement our clinical observations. However, we avoid strong conclusions about pathogen replacement or microbiological “stability,” as our data cannot definitively address these questions. Furthermore, microbiological findings should be interpreted with caution, given the exploratory nature of multiple pathogen-specific comparisons and the lack of adjustment for multiple testing.

Special mention should be made of Fusobacterium necrophorum, which, consistent with the literature, was found in our study to be associated with complications [22, 23]. While it was rarely isolated from ear cultures, it was relatively more prevalent in cultures obtained from subperiosteal or intracranial abscesses. Positive blood cultures of any pathogen, particularly Fusobacterium necrophorum, were significantly associated with higher complication rates. This observation echoes recent pediatric studies that identified Fusobacterium necrophorum as a major contributor to venous sinus thrombosis and other severe complications in AM [22, 23]. Specifically, Coudert et al. identified Fusobacterium necrophorum as the leading pathogen in sinus thrombosis complicating acute mastoiditis in a multicenter French pediatric cohort [22], and Yosefof et al. similarly reported it as the predominant organism in otogenic venous sinus thrombosis among Israeli children [23]. Our findings are consistent with these reports, further supporting the recognition of Fusobacterium necrophorum as a marker of severe complicated disease in pediatric mastoiditis.

From a public health perspective, our findings contribute to the growing body of evidence supporting judicious antibiotic prescribing for AOM. Among children who developed AM, we found no associations between prior antibiotic exposure and specific complication types. However, our findings are limited to complication severity among children who already developed AM and cannot inform whether delayed antibiotic treatment affects the risk of developing AM from AOM. Therefore, these findings should not be interpreted as direct support for delayed antibiotic prescribing strategies but rather as descriptive data on outcomes among children with established AM. This is particularly relevant given the substantial public health burden of antibiotic overuse. AOM represents a major contributor to antibiotic resistance globally, with antibiotic resistance identified by the World Health Organization as “one of the three greatest threats to human health” [24]. In the United States alone, more than 140,000 emergency department visits occur annually for antimicrobial-related adverse effects, comprising almost 20% of all drug-related adverse events [25]. Each excess day of antibiotic treatment has been associated with a 5% increase in the odds of antibiotic-associated adverse events [26]. Beyond direct toxicity, early-life antibiotic exposure disrupts the developing gut microbiome, with potential long-term consequences for immune development, metabolic health, and susceptibility to allergic and autoimmune diseases [27, 28].

The data from Thompson et al., which demonstrated that over 4,000 children would need to be treated with antibiotics to prevent a single case of AM [7], must be weighed against the finding that, among those who develop AM, early antibiotic treatment does not appear to mitigate the severity of complications. These findings are consistent with a more targeted approach to antibiotic treatment of AOM, though causal inference from observational data remains limited. However, it is crucial to emphasize that our findings do not address whether delayed treatment increases the risk of AM development—only that, once AM occurs, prior antibiotic exposure is not associated with protection against severe presentations. These observations support current AAP guidelines recommending selective antibiotic use based on patient age, symptom severity, and laterality [12], while underscoring the importance of parental education regarding warning signs that warrant urgent medical evaluation, including high fever, vomiting, severe headache, or pain behind the ear [29].

The retrospective, observational design creates substantial and likely insurmountable confounding by indication. Children who received antibiotics for AOM were selected for treatment by their community physicians based on clinical features that are themselves risk factors for severe disease progression. Critically, our dataset lacks the variables necessary to address this confounding. We do not have data on the severity of the initial AOM episode, the clinical trajectory before treatment decisions (symptom duration, response to antipyretics, parental assessment of severity), and the specific clinical reasoning behind antibiotic prescription decisions. Without these variables, we cannot use propensity score matching, instrumental variable analysis, or other quasi-experimental approaches to address confounding. While multivariable regression could adjust for measured confounders, we lack the key confounder—baseline AOM severity—that drives both treatment assignment and outcomes.

Furthermore, our study has several limitations that warrant careful consideration. Most critically, the retrospective, observational design introduces substantial risks of indication bias and unmeasured confounding. The lack of randomization means that patients in the two groups may differ in ways we cannot measure or adjust for, particularly with respect to initial disease severity at AOM presentation. Children who received antibiotics may have had more severe initial disease. This is a fundamental limitation of observational studies that cannot be fully addressed through statistical adjustment.

Measurement of antibiotic exposure is subject to several sources of misclassification. We relied on medical records, parental recall, and electronic community medical records to determine antibiotic use, with inherent limitations in capturing exact timing, dose, and adherence. The 48-hour cutoff, while clinically meaningful, is arbitrary and may misclassify some patients. However, sensitivity analyses using a 72-hour cutoff yielded even stronger associations (adjusted OR = 3.02 vs. 2.17), suggesting that misclassification would bias toward the null.

Although the events-per-variable ratio in the primary model was adequate (approximately 40), residual confounding by indication remains a fundamental limitation, as the key confounder, baseline AOM severity, was not captured in our dataset and therefore could not be included in the models.

Additionally, microbiological data were incomplete for some patients, limiting pathogen-specific conclusions. The study entry point was AM rather than AOM, so we cannot determine whether delayed antibiotic treatment affects the risk of progression from AOM to AM—only whether it affects complication profiles once AM has developed.

Despite these limitations, the study provides meaningful insights into the clinical presentation of AM in the context of modern antibiotic stewardship practices. While associations observed in our data suggest that prior antibiotic therapy does not prevent more severe complications in children who develop AM, the limitations discussed above preclude definitive conclusions about causality.

## Conclusion

This observational study examined associations between prior community-based antibiotic treatment and the presentation and outcomes of AM in children. Among children who developed AM, those who did not receive prior antibiotic treatment did not show increased rates of specific complications, such as subperiosteal abscess or intracranial complications, when hospitalized with AM.

Similar inflammatory markers, hospitalization durations, surgical intervention rates, and microbiological profiles across groups suggest that, once AM has occurred, complication severity is comparable regardless of prior antibiotic exposure. Importantly, this study does not address whether delayed antibiotic treatment affects the risk of developing AM from AOM, as this question requires comparing children with AOM who do versus do not progress to AM. Moreover, causal inference is limited by unmeasured confounding, and these findings should be interpreted as associations rather than causal relationships. Future studies are needed to better characterize the relationship between antibiotic timing and both AM development and complication severity. 

## Abbreviations

AOM: Acute Otitis Media
AM: Acute Mastoiditis
WBC: White Blood Cells
CRP: C-Reactive Protein
CT: Computed Tomography
MRI: Magnetic Resonance Imaging
SD: Standard Deviation
GAS: Group A Streptococcus
